# Eucapnic Voluntary Hyperpnea: Gold Standard for Diagnosing Exercise-Induced Bronchoconstriction in Athletes?

**DOI:** 10.1007/s40279-016-0491-3

**Published:** 2016-03-23

**Authors:** James H. Hull, Les Ansley, Oliver J. Price, John W. Dickinson, Matteo Bonini

**Affiliations:** 1 Department of Respiratory Medicine, Royal Brompton Hospital, London, UK; 2 National Heart and Lung Institute, Imperial College London, London, UK; 3 Department of Sport, Exercise and Rehabilitation, Faculty of Health and Life Sciences, Northumbria University, Newcastle upon Tyne, UK; 4 Carnegie School of Sport, Leeds Beckett University, Leeds, LS6 3QT UK; 5 Sports Therapy, Physical Activity and Health Research Group, School of Sport and Exercise Sciences, University of Kent, Chatham Maritime, UK; 6 Department of Public Health and Infectious Diseases, “Sapienza” University of Rome, Rome, Italy

## Abstract

In athletes, a secure diagnos is of exercise-induced bronchoconstriction (EIB) is dependent on objective testing. Evaluating spirometric indices of airflow before and following an exercise bout is intuitively the optimal means for the diagnosis; however, this approach is recognized as having several key limitations. Accordingly, alternative indirect bronchoprovocation tests have been recommended as surrogate means for obtaining a diagnosis of EIB. Of these tests, it is often argued that the eucapnic voluntary hyperpnea (EVH) challenge represents the ‘gold standard’. This article provides a state-of-the-art review of EVH, including an overview of the test methodology and its interpretation. We also address the performance of EVH against the other functional and clinical approaches commonly adopted for the diagnosis of EIB. The published evidence supports a key role for EVH in the diagnostic algorithm for EIB testing in athletes. However, its wide sensitivity and specificity and poor repeatability preclude EVH from being termed a ‘gold standard’ test for EIB.

**Electronic supplementary material:**

The online version of this article (doi:10.1007/s40279-016-0491-3) contains supplementary material, which is available to authorized users.

## Key Points

**Table:** 

Despite the long history and widespread use of the eucapnic voluntary hyperpnea (EVH) test in clinical practice, data to support its position as the ‘gold standard’ in the diagnosis of exercise-induced bronchoconstriction (EIB) in athletes are scarce.
The EVH test demonstrates poor test–retest reliability in athletes with mild EIB, and the implications for performance or health in an athlete with a 10–15 % fall in forced expiratory volume in 1 s following EVH still require elucidation.
The EVH test has a key role in diagnosing EIB in athletes but should not be termed the ‘gold standard’.

## Introduction

Exercise-induced bronchoconstriction (EIB) describes the phenomenon of transient reversible narrowing of the airways that occurs in association with exercise [1, 2]. The condition is highly prevalent in athletes, and establishing the diagnosis is important, given its potential impact on both health and performance [3, 4].

Research has consistently revealed a poor relationship between the presence of ‘asthma-type’ symptoms and objective evidence of EIB in athletes [5–8]. Furthermore, resting spirometric values are poorly predictive of EIB in this population [9]. Thus, to secure a diagnosis of EIB, it is important to perform objective testing to confirm any reversible change in airway function [10].

When an athlete’s history is suggestive of EIB, measuring the change in forced expiratory volume in 1 s (FEV_1_), before and following an exercise challenge test (ECT), represents the most intuitive method for diagnosis [1, 10, 11]. Indeed, since the 1970s, investigations have been conducted to standardize ECT procedures and interpretation of their results [12]. However, difficulties with ‘field’ exercise settings—specifically, inability to easily control ambient conditions and the challenge intensity—inherently limit the application of this approach [13]. Thus, whilst field-testing may be specific, it has poor diagnostic sensitivity for detecting EIB [14]. On the other hand, standardized laboratory exercise challenges may fail to properly reproduce the bronchoprovocative stimulus experienced by athletes when practicing in their own sporting discipline [15]. Moreover, minor alterations in exercise load and intensity can impact significantly on the prevalence of EIB [16].

Accordingly, a number of alternative or surrogate tests for diagnosing EIB have been proposed. These include indirect bronchoprovocation tests, which act to replicate the provocative airway stimulus induced by exercise and thus precipitate activation of the inflammatory cascade, causing airway smooth muscle contraction in susceptible individuals.

One particular indirect bronchoprovocation challenge, the eucapnic voluntary hyperpnea (EVH) test, has gained prominence in the diagnosis of EIB and it has long been recognized that hyperpnea of dry air provides a provocative stimulus to the airway [17–19].

The EVH test requires an athlete to complete a period of voluntary hyperpnea with a dry gas inhalant, which desiccates the airways, mimicking the osmotic priming stimulus to EIB [20]. The EVH methodology was established in 1984 to test army recruits for EIB [21] and has now been employed for the diagnosis of EIB in athletes for over 25 years [22].

Since the introduction of the EVH, its status as a precise and reliable test for the diagnosis of EIB in athletes has risen [23, 24]. Indeed, it is now often considered or cited as the ‘optimal’ means for establishing the diagnosis of EIB in athletes [25, 26]. Moreover, it is now frequently employed to ‘screen’ athletes for airway dysfunction [24, 27], as an inclusion criterion for studies [28], and to establish the efficacy of treatment interventions [29].

The aim of this article is to provide a state-of-the-art review of the place of EVH in testing athletic individuals for EIB. The review details EVH methodology and addresses key characteristics of EVH performance against other commonly utilized functional and clinical approaches in the diagnosis of EIB, as well as the influence on the response to the test of pharmacological and non-pharmacological interventions. The overall objective is to address the question, ‘does EVH really deserve to be considered a ‘gold-standard’ test in the diagnostic algorithm for EIB in athletes?’.

To achieve this aim, electronic searches were undertaken in the MEDLINE, ISI Web of Science, and The Cochrane Library databases. The registers were searched using the terms ‘eucapnic voluntary hyperpnea’, ‘eucapnic voluntary hyperpnoea’, ‘eucapnic voluntary hyperventilation’, and ‘EVH’ from the date of inception to July 2015. The search strategy yielded 612 articles (PubMed 200, ISI Web of Science 359, The Cochrane Library 53). Following the removal of duplicates, two independent reviewers selected papers of potential interest on the basis of titles and abstracts for a full-text assessment. Furthermore, reference lists of included studies, recent reviews, and textbooks were hand searched for relevant citations. This search resulted in 61 manuscripts that were considered relevant for the aim of this review.

## Background

Early work evaluating the clinical utility of hyperpnea as an airway challenge revealed the importance of maintaining isocapnia during the period of forced hyperpnea. This is important to avoid the deleterious clinical effects of systemic hypocarbia, but also given the fact that hypocapnia can promote bronchoconstriction [30].

The utility and clinical application of eucapnic hyperpnea was also initially limited by a belief that the cold air component of the stimulus was important and that continuous monitoring of end-tidal CO_2_ was required. Subsequently, Phillips et al. [31] demonstrated that the temperature of the air was less important and that a eucapnic balance could be maintained by admixing approximately 5 % CO_2_ in the inspirate. Eliasson et al. [22] also reported no difference in response between cold and dry air challenges.

In the 1980s, work in the Walter Reed Military Hospital, Washington, USA, led to the development of the modern-day protocol for EVH testing, which is most commonly utilized in athletes [20, 32].

## Eucapnic Voluntary Hyperpnea (EVH): Test Methodology

**Fig. 1:**
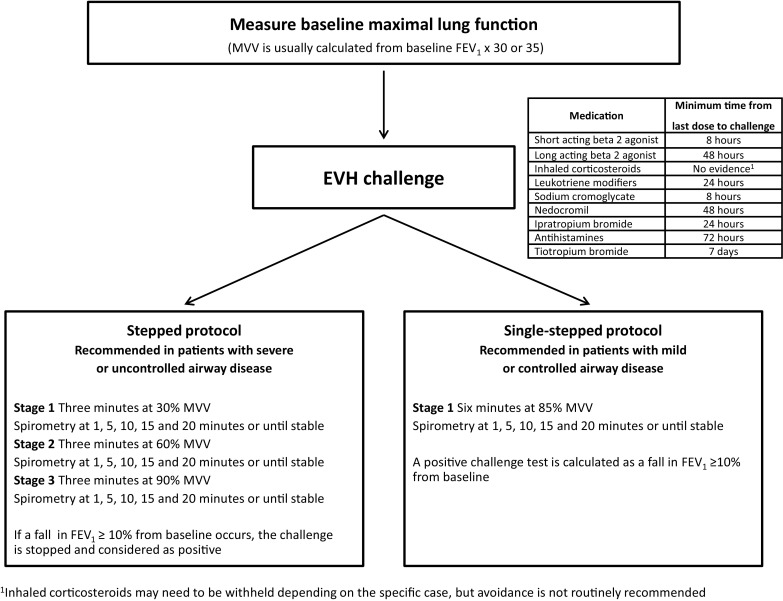
Eucapnic voluntary hyperpnea protocol and test recommendations. *EVH* eucapnic voluntary hyperpnea, *FEV*_*1*_ forced expiratory volume in 1 s, *MVV* maximum voluntary ventilation

Prior to undertaking an EVH challenge, subjects are required to adhere to the recommendations described in international guidelines for bronchoprovocation testing [11], including the duration for withholding inhaled asthma therapy (Fig. 1).

Subjects should be tested only when clinically well (i.e., free from a respiratory tract infection 6 weeks prior to the test) [33], should be advised not to ingest caffeine [34], and should not exercise on the day of the challenge, as this may exert a refractory protective effect against EIB [35].

Two types of EVH challenge methodology have been described: the single-stage and the stepped protocol (Fig. 1). The single-stage protocol is most commonly employed for athletes and requires subjects to maintain minute ventilation (*V*_E_) close to 85 % of their maximal voluntary ventilation (MVV) for 6 min [32]. The target ventilation is typically predicted by multiplying baseline FEV_1_ by 30 or 35 [36], although it is important to note that this approach is likely to be imprecise in elite athletes [37]. Thus, alternatively, target ventilation can be calculated from ventilation data obtained in a prior maximal aerobic exercise test [38].

During the challenge, it is crucial that participants achieve high (i.e., close to target) ventilation. Previous research has in fact demonstrated that halving the ventilation (e.g., only 15 × FEV_1_) and doubling the EVH challenge time resulted in a 60 % reduction in positive EVH challenge rate [32]. This has been reported to be because the small airways are excluded from the conditioning process, and hence the osmotic stimulus for bronchoconstriction is avoided [20]. Data from large cohorts of athletes and indeed clinical patients undertaking an EVH challenge actually suggest that while achieving a ventilation rate above 60 % of MVV (i.e., level that a test is considered valid) is physically challenging, it is readily achievable in most subjects [23, 39].

**Fig. 2:**
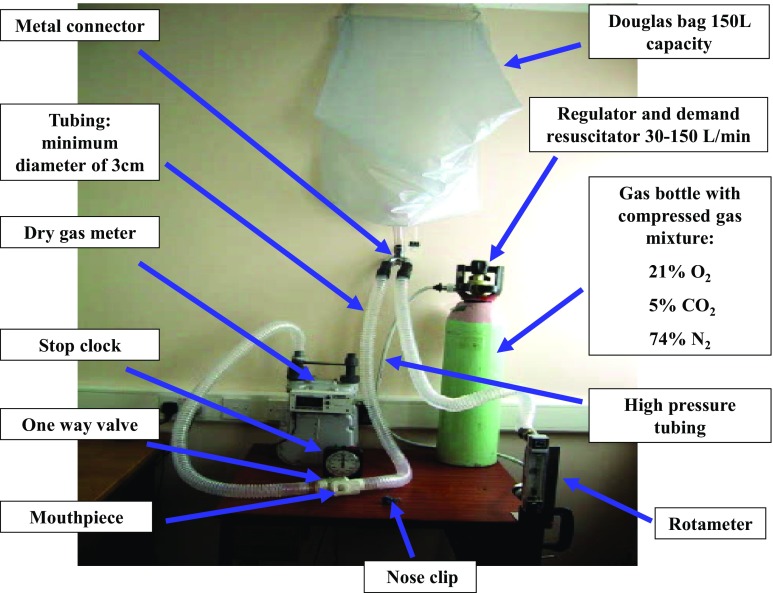
Photograph depicting the eucapnic voluntary hyperpnea challenge set-up

For a subject to achieve this target ventilation and simultaneously maintain eucapnia (i.e., perform hyperpnea), the compressed gas source should contain 21 % O_2_, 5 % CO_2_, with a balance of N_2_ (in the UK supplied as BOC code 280890 AK-PC). This inspirate can be administered from a gas cylinder via a direct demand valve or a Douglas bag/balloon (Fig. 2) or via a commercial system, which can be used to mix O_2_ and CO_2_ gases (EucapSys SMTEC, Switzerland). A video on how to perform an EVH challenge is available as online Electronic Supplementary Material (ESM) video S1.

Maximal flow volume loops are recorded at baseline and at several time points following the challenge [22, 39, 40] according to international guidelines for standardization of spirometry [41]. The greatest drop in FEV_1_ post EVH challenge (calculated as [pre − post FEV_1_]/[pre FEV_1_] × 100) is usually seen between 5 and 10 min post challenge [42].

Some authors have raised concerns regarding a possible clinical risk of EVH, in terms of its potential to precipitate a significant reduction in lung function [43]. However, our group has performed over 1000 EVH tests in athletes of a variety of disciplines, ages, and EIB severity (unpublished observations) without seeing any major adverse event such as a requirement for resuscitation, oxygen therapy, or hospitalization. Moreover, the EVH test has been reported to be well tolerated, even in a general population [44]. However, while acknowledging this, it has been proposed that the stepped protocol may be preferentially selected in individuals deemed to be potentially more susceptible to severe bronchoconstriction [32].

## Interpretation of the EVH Test

An EVH test is typically considered positive if the FEV_1_ falls ≥10 % from the baseline measurement within 20 min of challenge cessation [16, 28]. Indeed, some researchers have suggested that the ≥10 % FEV_1_ fall from baseline should be seen at consecutive time points post EVH and that the highest value out of two reproducible measurements (i.e., values within 150 ml or 5 % of each other) should be selected [1, 45]. Whilst this does support the physiological nature of a response, there are no robust data indicating that this criterion improves the sensitivity and/or specificity of the test.

The diagnostic threshold of a 10 % fall in FEV_1_ was initially derived from a study conducted in 90 asthmatic army recruits [41]. In this trial, a drop of 14 % was 100 % specific for asthma, but had a sensitivity of only 53 %. A threshold of 10 % was therefore recommended on the basis of an improved relationship between specificity and sensitivity (90 and 63 %, respectively). This value also aligns with the cut-off commonly employed in exercise studies [11]. An analysis of 860 athletes in 12 studies (Table 1) presents a mean drop of approximately 9 %, which is close to the current diagnostic threshold. However, the wide standard deviation (8.4 %) suggests that a lower threshold may be more appropriate. Indeed, whilst the ‘normative’ response to exercise is mild bronchodilation [46], it is our experience that the opposite is true following exposure to EVH.

**Table 1:** Studies reporting use of eucapnic voluntary hyperpnea in athletes

Study	Population	*N*	Total	Negative		Positive		Achieved ventilation (l/min)	Predicted ventilation (%)
			Fall in FEV_1_	%	Fall in FEV_1_	%	Fall in FEV_1_		
Holzer et al. [56]	Elite athletes	50	14.2 ± 15.5	50	3.0 ± 2.0	50	25.4 ± 15.0	126.8 ± 21.9	93.8 ± 4.7
Rundell et al. [14]	Elite winter athletes	38	9.1 ± 6.2	55	4.7 ± 3.2	45	14.5 ± 4.5	104 ± 26	82.6 ± 16
Dickinson et al. [27]	Winter athletes	14	13.6 ± 8.7	36	5.1 ± 2.5	64	17.0 ± 7.0	NR	NR
Parsons et al. [75]	College athletes	107	6.2 ± 2.6	61	4.2 ± 0.3	39	9.2 ± 0.1	NR	NR
Pedersen et al. [76]	Female swimmers	16	9.2 ± 7.9	67	5.2 ± 3.1	33	18.0 ± 8.4	NR	70.4 ± 13.0
Parsons et al. [77]	Non-asthmatic athletes	96	5.9 ± 4.3	82	NA	18	NA	NR	NR
Castricum et al. [73]	Elite athletes	33	13.2 ± 11.8	52	5.2 ± 2.7	48	21.6 ± 11.9	NR	EIB^+^: 78 ± 11 EIB^-^: 75 ± 9
Dickinson et al. [23]	Elite athletes	228	9.3 ± 9.8	66	4.6 ± 2.9	34	18.3 ± 11.9	NR	EIB^+^: 79.1 ± 11.2 EIB^-^: 79.5 ± 9.8
Ansley et al. [5]	Professional football players	65	14.0 ± 11.2	49	6.1 ± 2.8	51	21.5 ± 11.0	NR	EIB^+^: 74.7 ± 6.3 EIB^-^: 68.3 ± 10.1
Bolger et al. [78]	Summer sport female athletes	28	10.91 ± 7.15	64	5.8 ± 0.7	36	20.1 ± 2.5	NR	NR
Koch et al. [79]	Experienced male cyclists and triathletes	49	11.0 ± 9.0	71	8.0 ± 3.0	29	19.0 ± 14.0	NR	NR
Molphy et al. [39]	Recreational athletes	136	7.4 ± 6.7	87	5.4 ± 2.8	13	19.9 ± 9.7	NR	NR
Total		860	9.2 ± 8.4	67	5.1 ± 2.5	33	18.0 ± 9.8		

Data are presented as mean ± standard deviation unless otherwise noted

*EIB* exercise-induced bronchoconstriction, *EIB* ^*+*^ athletes with a positive EVH result, *EIB* ^*–*^ athletes with a negative EVH result, *EVH* eucapnic voluntary hyperpnea, *FEV*_*1*_ forced expiratory volume in 1 s, *NA* not applicable, *NR* not reported

**Fig. 3:**
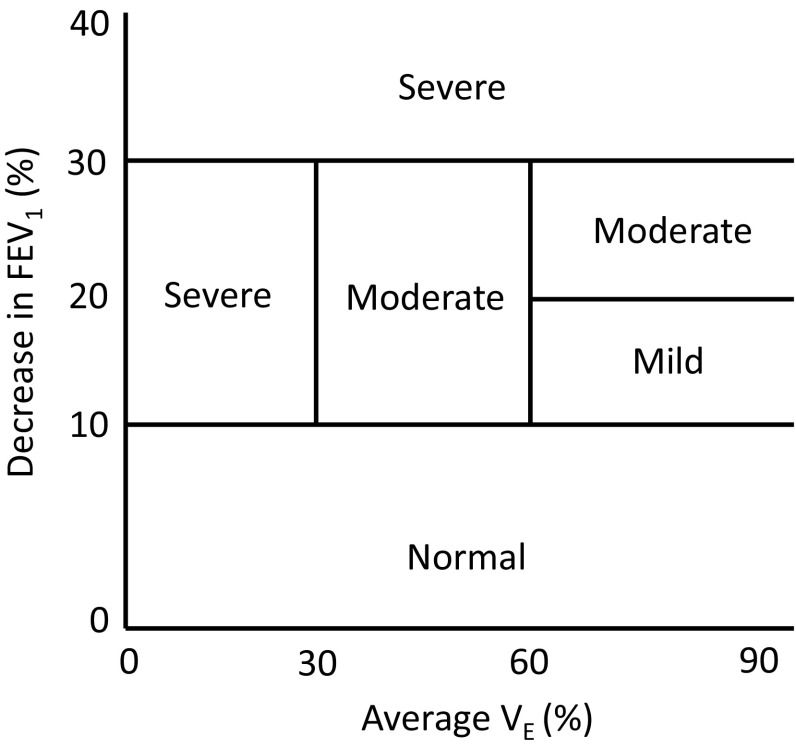
Degree of bronchoconstriction after a eucapnic voluntary hyperpnea challenge in relation to the FEV_1_ % fall compared with baseline and the ventilation rate maintained during the test *FEV*_*1*_ forced expiratory volume in 1 s, *V*_*E*_ minute ventilation

The fall in FEV_1_ during a bronchoprovocation challenge is dependent on the level of ventilation maintained during the test [20, 32, 47]. In light of this, it is important to report the *V*_E_ achieved during the test (Fig. 3). It has been proposed that the severity of bronchoconstriction following an EVH test can be classified as mild (≥10 to ≤20 %), moderate (≥20 to ≤30 %), or severe (>30 %), depending on the magnitude of the largest drop in FEV_1_ and ventilation achieved [20].

The EVH test appears to have good test–retest reproducibility when a subject develops airway narrowing of at least moderate severity (i.e., >20 %) [48, 49]; however, it appears to demonstrate poor repeatability at levels approximating a mild fall in FEV_1_ (i.e., 10–15 %). Indeed, in a recent study by Price et al. [50], the poor diagnostic repeatability of EVH indicated that, in a cohort of recreational athletes with a mild reduction in FEV_1_ post EVH, clinicians should not depend on a single positive test to support or refute a diagnosis.

The role and value of other surrogate physiological measures of airflow and bronchoconstriction following EVH have yet to be established. Indeed FEV_1_ was found to be more accurate than forced expiratory flow at 50 % (FEF_50_) and, although PEF displayed a similar association, this parameter is highly effort dependent and thus intrinsically less reliable [51]. Other indices of airway dysfunction (i.e., those obtained by impulse oscillometry) have the potential to provide important information following EVH [52, 53], yet their contribution in this context remains to be fully established.

## Comparison of EVH with Other Diagnostic Assessments

A key determinant of whether EVH deserves to be termed a diagnostic ‘gold standard’ centers on whether it measures and detects the condition of interest, i.e., does it reliably diagnose EIB? To evaluate this, it is important to consider how the diagnosis of EIB is best established and thereafter how EVH compares with other diagnostic assessment tools.

In medical practice, the performance of a diagnostic test is usually considered against an approach based on clinical assessment, i.e., via history and examination. This clinical assessment would typically focus on the detection of symptoms such as exercise-associated cough, wheeze, and breathlessness. However, in athletic individuals, the standard clinical diagnostic approach to assessment has been proven unreliable. This disconnect between symptom perception and bronchospasm is highlighted in recent studies [54, 55], including the work by Simpson et al. [55] that describes how athletes continued to report multiple symptoms despite successful attenuation of airway narrowing with beta-2 agonist treatment.

Thus, as might be expected, a great number of studies have highlighted a poor predictive value of respiratory symptoms for diagnosing EIB. Holzer et al. [56] studied 50 elite summer-sport athletes: of the 42 subjects reporting one or more asthma symptoms, only 25 had a positive EVH challenge result. The symptoms with the greatest positive predictive value for the EVH challenge were wheeze without a cold, night awaking with chest tightness, and exercise-associated dyspnea. Similarly, Dickinson et al. [23] evaluated 228 British athletes and showed that, among the 30 athletes reporting a history of asthma, nine (almost one-third), were EVH negative. In those with a positive response to EVH (*n* = 78; 34 %), only 21 had a previous ‘clinical’ diagnosis of EIB. Ansley et al. [5] demonstrated similar findings in professional football players. In fact, of the 65 players assessed, clinical symptoms during exercise were reported by 57 (88 %) athletes, despite only 20 of 42 having a positive EVH response.

The EVH challenge has also been assessed against alternative objective tests utilized in the diagnosis of EIB. It is intuitive to compare the test with an exercise challenge, and this was comprehensively reviewed by Stickland et al. [57]. Their systematic analysis of EVH versus ECT included seven prospective cross-sectional trials. The number of participants in each study ranged from 10 to 33 subjects. Four studies were clearly performed in elite athletes, whilst three did not report the level of fitness or sports participation.

From a combined total of 138 participants studied, 42 (30 %) tested positive for EIB with the ECT and 74 (54 %) tested positive with the EVH. However, overall EVH sensitivity and specificity were extremely variable, with values ranging from 25 to 90 % and from 0 to 71 %, respectively. In studies enrolling only athletes, ranges of sensitivity and specificity only narrowed marginally from 25 to 88 % and from 0 to 67 %, respectively. Using a different diagnostic threshold for a positive result, the sensitivity improved at a FEV_1_ % fall ≥15 %, yet considerable variability remained among the studies.

The authors concluded that methodological issues, such as EVH protocols employed and populations studied, limited the interpretation and generalizability of results. They also raised concerns regarding a significant risk of spectrum bias, i.e., potential for the performance of a diagnostic test to vary in different clinical settings because each setting has a different representation of subjects.

Sue-Chu et al. [58] compared airway hyper-responsiveness (AHR) to methacholine, EVH, exercise, adenosine 5′-monophosphate (AMP), and mannitol in 58 cross-country skiers. Bronchial hyper-responsiveness was detected in 23 subjects to methacholine (39.6 %), in five subjects to AMP (8.3 %), and in three subjects to mannitol (5.1 %). A total of 25 (43 %) subjects were hyper-responsive to at least one of these stimuli. Of the 33 athletes tested, three (9 %) and six (18 %) were hyper-responsive to EVH and field exercise tests, respectively. In those with a negative methacholine challenge, bronchial reactivity to either stimulus was detected in four subjects, while no subject was positive to both tests. On the other hand, of the 14 (42 %) skiers with methacholine hyper-responsiveness, three were hyper responsive to either test and one to both tests.

The sensitivity of a challenge with mannitol to identify responsiveness to EVH has been assessed in 50 elite summer sport athletes [59]. A total of 27 subjects were previously diagnosed with asthma by a doctor, and 21 were currently under treatment for EIB or asthma; 25 athletes were positive to EVH, and 26 subjects had a positive (provoking dose causing a 10 % fall in FEV_1_ [PD10]) mannitol challenge. Mannitol demonstrated a sensitivity of 96 % and specificity of 92 % to identify a positive response to EVH, prompting the authors to suggest mannitol as a valid alternative to identify EIB.

More recently, 24 summer-sports athletes who reported respiratory symptoms on exertion performed a standard EVH and a mannitol challenge on separate days [60]. Of these, 11 (46 %) showed a sustained ≥10 % FEV_1_ fall after EVH, while eight (33 %) were positive (15 % FEV_1_ fall) to mannitol. A strong association was found between the two tests (*r* = 0.7, *P* < 0.001).

Finally, Osthoff et al. [61] assessed the feasibility of an EVH challenge against mannitol to detect AHR in elite athletes with disability. Among the 44 athletes studied, nine (20 %) were positive to EVH, and eight (18 %) were positive to mannitol (PD10); 14 (23 %) subjects were positive to at least one challenge, and only three athletes were positive to both. The EVH test showed better positive and negative predictive values to detect physician-diagnosed asthma compared with mannitol (89 and 91 % vs. 75 and 86 %, respectively).

Overall, therefore, it is apparent from these findings that there can exist significant discrepancies between the response to an EVH test and clinical symptoms, as well as the results of different ‘provocation’ challenges.

## Influence of Pharmacological and Non-Pharmacological Interventions on EVH Response

The EVH test is also often employed to objectively evaluate airway response to a therapeutic intervention. A single dose of terbutaline (0.5 mg) has been shown to offer significant protection against hyperpnea-induced bronchoconstriction [62]. Moreover, Kippelen et al. [63] reported that a single high dose of beclomethasone dipropionate significantly inhibited bronchoconstriction after EVH in both untrained subjects and athletes with EIB. The same group showed a significant bronchoprotective effect against EVH with sodium cromoglycate [64]. In a randomized, double-blind, placebo-controlled trial performed in 11 physically active EIB positive subjects, montelukast provided 44 % protection from EIB after EVH [65].

The impact of non-pharmacological interventions on EVH response has also been evaluated, particularly in the context of dietary modification. Tecklenburg-Lund et al. [66] showed that a 3-week fish oil supplementation significantly inhibited hyperpnea-induced bronchoconstriction.

The effect of a patented marine lipid extract (PCSO-524) was more recently assessed in a double-blind randomized controlled trial (RCT) performed in 20 subjects with asthma [67]. Results obtained showed that the PCSO-524 diet significantly reduced the maximum FEV_1_ fall post EVH compared with usual and placebo diet.

In a double-blind, placebo-controlled RCT in 18 EIB-positive subjects, the post-EVH bronchoconstriction was significantly attenuated after 4 and 8 weeks of supplementation with whey proteins [68].

## Discussion

### Is EVH Truly a ‘Gold Standard’ for the Diagnosis of Exercise-Induced Bronchoconstriction?

The term ‘gold standard’ is generally taken to represent the ‘paradigm’ of absolute correctness and the best standard in the field against which to compare the characteristics of a novel diagnostic procedure or method [69].

The EVH test has been cited as optimal or the ‘gold standard’ and employed in clinical practice to provide a definitive diagnosis of EIB in athletes presenting with respiratory symptoms [3]. As such, it has been endorsed by the International Olympic Committee as the airway challenge of choice in the diagnosis of EIB in athletes [20].

Our review has highlighted that, despite the long history and widespread use of EVH, data to support its position as the ‘gold standard’ are sparse. Indeed, within the limited dataset available, the data are highly heterogeneous with regards to athletes’ age, sex, sporting discipline, and level of physical activity. In addition, there are significant methodological differences concerning the clinical (EIB with or without asthma) and functional (FEV_1_ % fall) adopted diagnostic criteria, the length of the challenge, and the ventilation rate, to permit a definitive conclusion.

The comprehensive systematic review comparing EVH with ECT performed by Stickland et al. [57] indicates that even the best studies reveal a variance in specificity and sensitivity to a degree that cannot permit the term ‘gold standard’ to be applied. Similar conclusions can be reached analyzing studies assessing the role of EVH against other direct and indirect challenges (e.g., mannitol).

Moreover, in terms of methodology, the EVH test has important limitations, including the cost of the compressed gas mixture and a requirement for specialist equipment and skilled technicians to conduct the challenge. In this respect, trained specialists should perform EVH, with precautions taken to minimize the risk of an adverse event (i.e., severe bronchoconstriction) [1].

It could be also debated whether EVH represents an appropriate diagnostic test only in athletes or even in non-competitive exercisers and sedentary subjects who might not easily maintain high ventilation rates for a prolonged time. Indeed, one of the major strengths of EVH testing in elite athletes is the ability to achieve a V_E_ rate that mimics the demands of high-intensity exercise. However, when considered from a recreational or sedentary perspective, the increased ventilation associated with EVH may not reflect real life, i.e., EVH may desiccate the airway to a greater extent than typical exercise in normal subjects.

This observation may be particularly pertinent in the evaluation of airway symptoms in elite swimmers. In this specific population of athletes, there exists a high prevalence of respiratory symptoms [70], and EVH has been utilized to characterize heightened AHR [71, 72]. Yet, the obvious differences that exist between the dry, cold stimuli of EVH and the humid, moist, warm environment encountered in the pool are substantiated by comparator studies indicating a significantly lower prevalence of EIB from testing performed at the poolside [73].

Despite these limitations in respect to the application of the term ‘gold standard’, it remains the authors’ opinion that EVH is a valuable indirect bronchoprovocation test in the context of testing athletes for EIB. Moreover, we believe that the key utility of the EVH test lies with the finding of a negative result, i.e., in terms of the ability to rule out a diagnosis of EIB. However, it is equally important that prevalence estimates are accurate and not over-estimated by the application of overly sensitive diagnostic test methodologies, i.e., resulting in a false-positive diagnosis and potential for mistreatment. Nevertheless, it is essential that clinicians continue to utilize and undertake some form of objective testing to establish a secure diagnosis of EIB and not to rely on symptomatic assessment alone.

### Unmet Needs and Future Perspectives

Several questions regarding the use of EVH in athletes need to be answered by innovative and well-designed research studies.

First, the ‘standard’ against which to compare the EVH challenge for the diagnosis of EIB, remains unclear. Whilst a clinician-based diagnosis is often used in ‘asthma’ studies, this is clearly not appropriate in studies of EIB in athletes. It is not appropriate to nominate EVH to be the ‘gold standard’ on the basis that is it most likely to be positive and thus may be the most sensitive test. The literature mostly refers to either field or laboratory exercise challenges; however, study designs are heterogeneous, and some utilize logical protocols with dry gas as an inhalate, while others are less well controlled [74]. Very little robust evidence is available regarding relationships with other potentially valuable ‘diagnostic’ endpoints, i.e., if the aim of EIB detection is to facilitate delivery of treatment to mitigate symptoms (e.g., exercise dyspnea, cough, and wheeze), then a more logical comparator may actually be longitudinal therapeutic response. Likewise, if the aim is to ensure optimal performance, then use of performance endpoint or surrogate may be more logical. In the future, alternative surrogates of airway inflammatory patterns (e.g., periostin levels) may also become relevant comparators. Thus, it may be that long-term follow-up and surveillance of clinical, physiological, and inflammatory markers is required to determine whether EVH ‘predicts’ response to treatment and whether it is of support to manage airway health. It may also be appropriate to evaluate outcomes in the context of athletic performance [4].

On the other hand, findings relating EVH to direct bronchoprovocative challenges are likely to be negatively influenced by the poor predictive value of these procedures for detecting EIB in athletes. Therefore, to properly assess the reliability of EVH in diagnosing EIB in athletes and to evaluate the best standard in this context, appropriate comparators and endpoints need to be considered and agreed.

It is unclear whether EVH provides the same reliability, in terms of sensitivity and specificity, for diagnosing and distinguishing between EIB with and without underlying clinical asthma, which according to the most recent international guidelines should be considered two distinct phenotypes in view of the different pathophysiologic mechanisms and inflammatory patterns [10].

There is also a need to re-evaluate and investigate what is an appropriate ‘cut-off’ value for EVH, as well as to better standardize the methodology in performing the test. It is not clear whether a 10 % fall in FEV_1_ really represents the most appropriate diagnostic threshold. The EVH test demonstrates poor test–retest reliability in athletes with mild EIB, and the implications on performance or health for an athlete with a 10–15 % fall in FEV_1_ following EVH still require elucidation.

## Conclusion

The EVH test has a key role in the diagnostic algorithm for EIB testing in athletes. It undoubtedly detects moderate to severe AHR in susceptible athletes, and its greatest value appears to lie in its negative predictive value. However, the wide sensitivity and specificity indices and poor repeatability in mild to moderate cases preclude EVH being termed a ‘gold standard’ test for EIB.

## Electronic supplementary material

Below is the link to the electronic supplementary material.

**Video file 1.** Video of eucapnic voluntary hypopnea challenge test being performed (MP4 2470 kb)
